# Simulation and Validation of Porosity and Permeability of Synthetic and Real Rock Models Using Three-Dimensional Printing and Digital Rock Physics

**DOI:** 10.1021/acsomega.1c04429

**Published:** 2021-11-16

**Authors:** Ezdeen R. Ibrahim, Mohamed Soufiane Jouini, Fateh Bouchaala, Jorge Gomes

**Affiliations:** † Department of Geosciences, Khalifa University, Abu Dhabi 127788, United Arab Emirates; ‡ Department of Mathematics, Khalifa University, Abu Dhabi 127788, United Arab Emirates; § Abu Dhabi National Oil Company, Abu Dhabi 898, United Arab Emirates

## Abstract

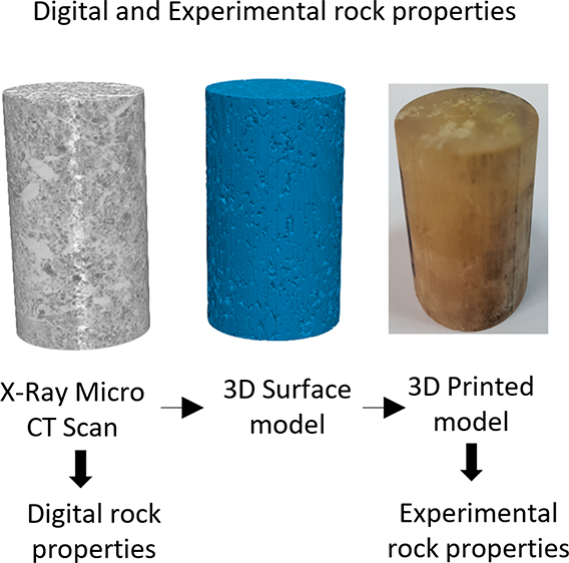

A standard digital rock physics workflow aims to simulate petrophysical properties of rock samples using few millimeter size subsets scanned with X-ray microtomography at a high resolution of around 1 μm. The workflow is mainly based on image analysis and simulation procedures at a subset scale leading to potential uncertainties and errors that cannot be quantified experimentally. To overcome the gap between scales, we propose to integrate three-dimensional (3D) printing technology to generate enlarged subsets at a scale where experimental measurements are feasible to validate simulated results. In this study, we 3D printed synthetic and real samples and compared digital and experimental rock properties. The most challenging phase in the workflow consists of the difficulties encountered while cleaning the 3D printed samples to remove the support material. Results for subsets extracted from synthetic, sandstone, and carbonate samples showed good agreement between digital and experimental measurements for porosity values less than 12% and a range of permeability values between 100 and 2000 mD.

## 1 Introduction

**Figure 1:**
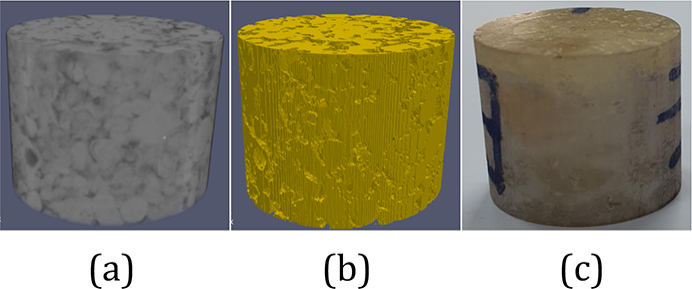
(a) Original 3D X-ray microtomography of the cylindrical 2 mm diameter subset, (b) solid-phase surface extracted after image segmentation, and (c) 3D printed sample enlarged to a diameter of 38 mm.

Digital rock physics (DRP) aims to better characterize rock properties of oilfield reservoir samples using X-ray microtomography images and numerical simulations. The general workflow includes three main steps consisting of image acquisition, image segmentation, and numerical simulation. The image acquisition is based on scanning of a standard 3.8 cm cylindrical core plug at a coarse resolution (around 20 μm) in order to have a general overview of the sample heterogeneities. Then, smaller subsets of few millimeter size are physically extracted and scanned at a fine scale (around 1 μm) to characterize the pore network. DRP uses these high-resolution representations of the pore network to simulate several rock properties such as porosity, permeability, and elastic moduli. At the image acquisition step, the resolution of the scanned image is constrained by the sample size.^1−3^ Thus, if we need to image a sample at a fine scale, then we need to extract physically a few millimeter size subset and then scan it. Several DRP studies in sandstone reservoirs showed that fine-scale simulations on few millimeter subsets considered as a representative element volume (REV) provide simulated rock properties in good agreement with experimental properties measured at a coarse scale.^4−8^ However, this type of approach reaches its limitation in carbonate rocks due to their heterogeneities.^9−11^ For carbonate reservoir rocks, the general workflow includes an additional step consisting of characterizing subsets at a fine scale representative of the various textures visualized at a coarse scale. An upscaling procedure is applied, then, to simulate the effective rock properties.^12−17^ Validation is usually obtained by a comparison with the experimental rock properties obtained at the coarse scale. However, the local properties obtained at a fine scale by simulation are not validated experimentally because of their small size. In this study, we propose to study the feasibility of using 3D printing to enlarge fine-scale subset images to a size that allows for experimental studies and hence the one-to-one validation of simulated results (Figure 1).

In the last decade, several studies investigated the ability of 3D printing technology to enhance the characterization of rock properties for petroleum engineering and geoscience applications. Indeed, 3D printing has the main advantage of replicating realistic digital models obtained through image acquisition devices into real physical samples on which experimental laboratory measurements can be performed. These models can be used as a complement to the digital rock physics approach to better characterize rock properties such as petrophysical and mechanical properties by creating analog models even for highly complex geometry samples.^18,19^ Furthermore, the diversity of resin materials available helps to model natural rock behavior under several types of experimental conditions.^20^

The accuracy of 3D printing techniques in replicating complex pore network geometries depends on several factors such as the 3D printing technology, the type of resin used, and the post-processing. In this paper, we focus on two technologies to replicate porous media representing rock samples of an oilfield reservoir. Multijet printing (MJP) is a 3D printing procedure using piezo printhead technology. The process is based on depositing layer-by-layer photocurable plastic resin or casting wax materials.^21^ The MJP 3D printing technology can create models with fine feature details at a resolution reaching 15 μm in the vertical direction. Another advantage of using this type of technology is that the used support material is dissolvable at a certain range of temperatures without damaging the main solidified structure. Stereolithographic (SLA) 3D printing technology implements curable resins, which are photopolymerized layer by layer using a laser beam to create a 3D model.^22^ The mechanical behavior of 3D printed models using SLA technology mainly depends on the chemical composition of the curable used resin. There are several types of SLA resins such as oligomers, monomers, and photoinitiators.^23^ The main parameters controlling the mechanical properties of the 3D printed sample during the procedure are temperature, pressure, and humidity. Indeed, a higher pressure and temperature generate a stiffer material, whereas a higher humidity reduces surface stiffness.^20,21^

Several studies adopted 3D printing technology to generate replicas of real rock samples for destructive tests keeping intact the original samples.^24−26^ The use of 3D printing can also improve the experimental repeatability of laboratory measurements reducing the statistical experimental errors.^27,28^ Furthermore, 3D printing was implemented to experimentally study the transport property changes of the rock microstructure due to compaction and dissolution processes.^29^ In recent studies, 3D core samples have been printed with different materials and printing technologies to assess the petrophysical properties of the replicas in sandstone reservoir rocks.^26,30,31^ For example, several 3D printing techniques were used to create rock analogs of real heterogeneous samples in order to assess uniaxial and triaxial compressive measurements.^19^ Quantitative measurements showed that strength and deformation characteristics of printed samples were comparable to real data. This agreement reveals the great potential of 3D printing technology to be used for more complex rocks such as carbonates. The integration of 3D printing as a tool for the experimental validation of simulations, obtained at a fine scale, could improve the reliability of upscaling procedures for carbonate rocks.

## 2 Methodology

In this study, we propose a workflow that consists of four main steps. First, we scanned 38 mm diameter cylindrical samples at a coarse scale (20 μm resolution). Then, we extracted and scanned subsets of few millimeter size at a fine scale (1 μm resolution) using X-ray computed tomography scanners. We implemented image analysis to segment subsets scanned at a fine scale and extracted 3D surfaces representing the solid phase. Second, we simulated porosity and permeability numerically into subsets imaged at a fine scale. Third, we printed in 3D the enlarged subsets into a scale at which we can run experimental measurements. Fourth, we experimentally measured rock properties and compared them to the simulated ones.

### 2.1 Image Acquisition and Segmentation

**Figure 2:**
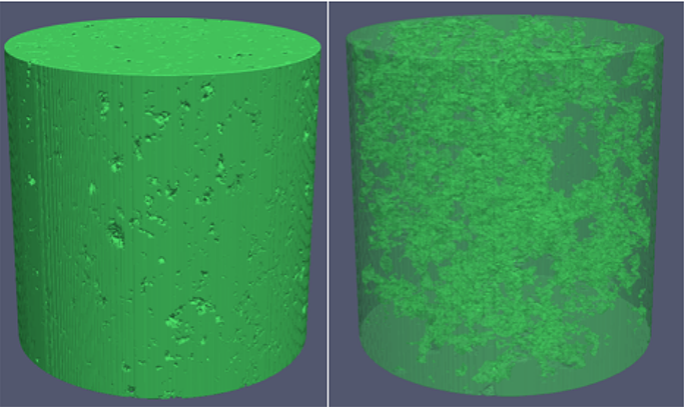
Surface of solid phases extracted from two carbonate subset samples.

The first step of the workflow consists of using an X-ray microcomputed tomography scanner to characterize rock samples at coarse and fine scales. The microtomography scanning system consists of three elements, which are the source, the detector, and the sample. The acquired image is a 3D block of voxels representing gray levels derived from the X-ray attenuation and related directly to the rock density. We have used a Zeiss Xradia X-ray microcomputed tomography scanner, available in our research center, which has a detector size of 2100 × 2100 pixels and a field of view of 1 to 100 mm. At the coarse scale, we obtained an image revealing the main heterogeneities present in a sample. Then, we selected homogeneous cylindrical zones of 1 to 3 mm diameter and extracted them physically.^17,32^ We scanned these subsets at a fine scale of around 1 μm resolution to capture the pore network. At this stage, several image segmentation techniques can be implemented to extract the pore network from the solid phase based on manual, semiautomatic, or fully automatic approaches. The choice of the segmentation technique mainly depends on the complexity of the 3D image in terms of gray level distribution.^3^ In our study, we implemented the Otsu’s method for very simple cases with clear separation between pore and grain gray level distribution.^33^ For more complex samples revealing an overlap between solid and porous phases’ gray level distribution, we implemented *K*-means and bi-level segmentation methods.^34^ The segmentation output result is a 3D binary image denoting the pore and grain positions in the scanned sample. This result is subsequently used for extracting the 3D surface representing the solid phase to be used as a digital model for 3D printing (Figure 2). Then, we use the digital model for the numerical estimation of the porosity and the permeability properties. Furthermore, we used an X-ray computed microtomography acquisition system to scan the 3D printed samples to verify the printer accuracy in representing the rock digital models. Eventually, another important use is to scan the 3D printed samples after the cleaning process to verify the cleaning performance in removing the support material.

### 2.2 Numerical Simulations

We simulated permeability using the lattice Boltzmann method (LBM). The LBM incorporates the Bhatnagar–Gross–Krook (BGK) model, which defines a fluid as a set of particles that collide and stream. The velocity of each particle is computed iteratively using only the nearest neighbor velocities, and the absolute permeability is provided through Darcy’s law based on the overall average flux.^35,36^ This is governed by the following distribution function
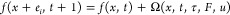
1where *x* and *t* denote the time and location of a particle, respectively, *e_i_* is the particle velocity in the *i*th direction, τ is the relaxation, Ω is a collision operator, and *F* is an external force. Equation 2 computes the momentum density of a particle located in position *x* at time *t*
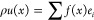
2where ρ represents the density of the fluid.

Finally, the absolute permeability is computed using Darcy’s law
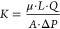
3where *K* is the absolute permeability, *Q* is the average velocity of particles, Δ*P* is the gradient of pressure along a sample of length *L*, μ is the fluid viscosity, and *A* is the surface area of the sample cross section.

The main advantage of using this approach is that velocity is computed at each iteration using only the nearest neighbor velocities, which makes it ideal for massively parallel computers.

The physical permeability is obtained as follows
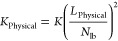
4where *L*_Physical_ is the sample edge length in SI units and *N*_lb_ is the number of lattice spaces along an edge. The ratio  represents the image resolution.^37^

The simulated physical permeability results, for subsets imaged at a fine scale, are compared to experimental laboratory measurements obtained for 3D printed enlarged samples.

### 2.3 3D Printing and Experimental Measurements

In this study, we used two types of 3D printers: a ProJet MJP 3600 from 3D Systems and a Form 2 from Formlabs. The ProJet MJP 3600 is based on multijet printing (MJP) technology, which is a material jetting printing process that uses piezo printhead technology to deposit materials layer by layer. The latter has an equivalent horizontal and vertical resolution of 30 μm. The resin properties required for the ProJet MJP 3600 printer and the support material are summarized in Table 1. The Form 2 printer uses stereolithography (SLA) technology based on layer-by-layer printing, by using photochemical processes where a laser beam solidifies chemical monomers to form polymers. The Form 2 3D printer uses a photoreactive resin to produce samples with vertical and horizontal resolutions of 25 and 100 μm, respectively. The properties of the adequate resin used for Form 2 3D printing and the support material are reported in Table 1. The printing time for an STL file mainly depends on the printer resolution. In our case, the printing of a 38 mm diameter and height cylindrical sample with a vertical resolution of around 25 μm took 12 h.

**Table 1:** Resin Properties

	ProJet MJP	Form 2
density of liquid (g/cm^3^)	1.02	1.25
distortion temperature (°C)	56	58
tensile strength (MPa)	42.4	64.67
tensile modulus (MPa)	1463	2771
elongation at break (%)	6.83	6.21

The main input for the two 3D printers is the 3D digitized and meshed surface of the solid phase, usually saved in a stereolithography format (STL).

### 2.4 Cleaning and Experimental Measurements

**Figure 3:**
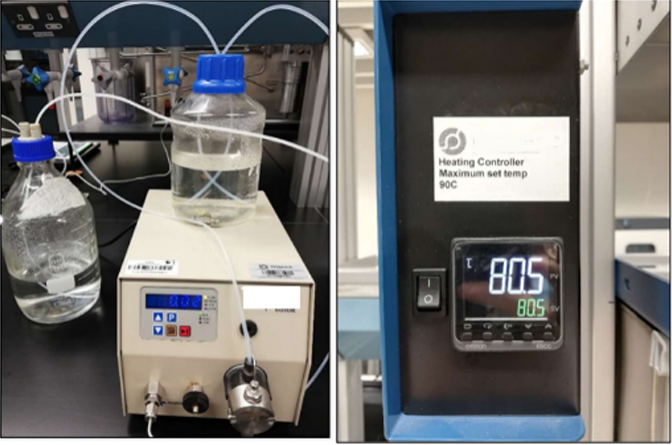
Pump supplying distilled water as a flushing fluid with a flow rate of 0.02 cc/min and at a temperature set to 80 °C.

**Figure 4:**
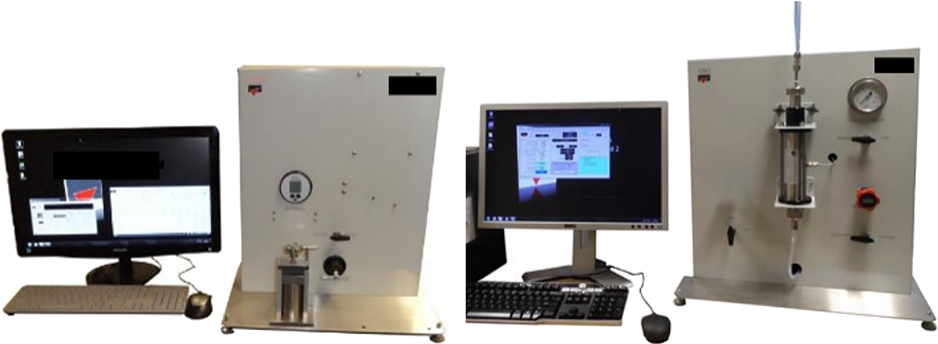
Vinci Technologies helium porosimeter and a steady-state gas permeameter, from left to right, respectively.

The challenging step in the proposed experimental procedure is the cleaning of the 3D printed models to ensure the removal of all support material (soft resin) blocking the pore networks. To achieve this goal, we placed the samples in an oven at 80 °C, and then, we flushed them with distilled water at around 80 °C and a flow rate of 0.02 cc/min, a confining pressure of 400 psi, and a backpressure of 70 psi. Backpressure is crucial to ensure opening of the whole pore network (Figure 3). The pump was set to stop if the pore pressure exceeds half of the confining pressure (200 psi). Cleaning was done over a period ranging between one to two weeks depending on the sample. Cleaning was considered completed when the output fluid at the outlet became clear, indicating that all support material was removed. Then, we scanned the 3D printed samples to verify the cleaning performance. Finally, we conducted poroperm measurements using a Vinci Technologies helium porosimeter and a steady-state gas permeameter (Figure 4).

## 3 Results and Discussion

In order to validate the proposed procedure, we initially generated four synthetic 3D surfaces representing helical tube samples with different tube radius sizes and tortuosity values (S_1_ to S_4_). Four cylindrical samples of 38 mm diameter and height were generated by using the two 3D printers. The main advantage of having these synthetic samples is the simplicity of the cleaning procedure and the availability of an analytic solution to validate the absolute permeability, in addition to the simulation and experimental measurements. The analytic solutions of absolute permeability for samples with helical tube pore networks is given by
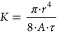
5where *K* is the permeability, *A* is the cross-sectional area, *r* is the radius of the helical tube, and τ is the tortuosity.

**Figure 5:**
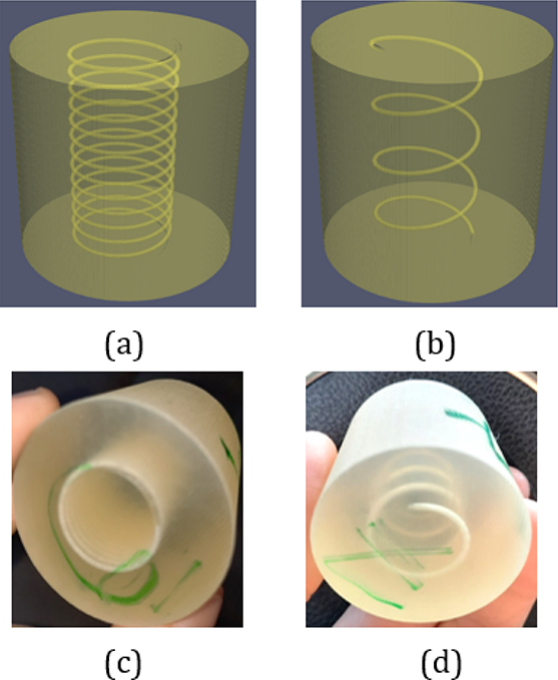
Helical tubes with two different tortuosity values: (a,b) digital models and (c,d) 3D printed samples.

Experimental laboratory measurements obtained from the two 3D printers are similar for the simple helical tubes. We report in Table 2 the experimental results obtained by using the ProJet 3D printer and compare them to the digital values. The experimental porosities are in very good agreement with the digital ones obtained from the segmentation technique. This agreement validates the cleaning and the 3D printing process, which was able to correctly capture the helical tubes generated with different tortuosity values (Figure 5). The experimental permeability is also in good agreement with both analytic and LBM simulations values.

**Table 2:** 3D Printed Samples of Helical Tubes: Experimental and Simulated Porosity and Permeability Using the ProJet 3D Printer

	porosity (%)		permeability (mD)		
	digital	experimental	digital	analytic	experimental
S_1_	1.0	1.2	84	95	124
S_2_	0.4	0.4	156	163	145
S_3_	2.3	2.2	191	209	327
S_4_	1.5	1.4	352	378	517

**Figure 6:**
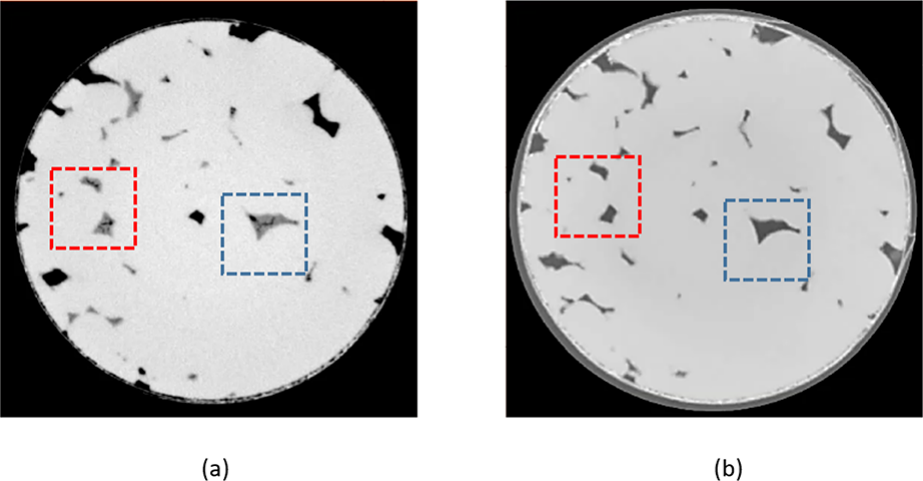
Cleaning process efficiency for sample S_5_. (a) Before cleaning: intermediate gray level values illustrating the soft resin trapped inside pores highlighted in red and blue and (b) after cleaning: gray levels changing to zero (black) corresponding to air.

Then, we generated six other samples based on a variety of real rock sample models originating from sandstone and carbonate reservoirs. For all these samples, we applied the described workflow consisting of image acquisition, numerical simulation, 3D printing, cleaning, and experimental measurements. Samples S_5_ and S_6_ were generated from 3D X-ray images of two homogeneous sample subsets of carbonate rocks. The digital porosity using image segmentation gave an estimated value of 12.1% for the sample S_5_ and 11.6% for the sample S_6_. Also, we numerically simulated the permeability using the LBM and converted the permeability from LBM units to physical units using eq 4. We found a value of 39.5 D for the sample S_5_ and 49.8 D for the sample S_6_. Both values are beyond the maximum sensitivity of the measurement experimental setup of 10 D. Thus, these two samples were only used for porosity validation. Experimental laboratory porosity measurements gave 12.3% for the sample S_5_ and 11.5% for the sample S_6_. Porosity results are in high agreement with digital result estimations, which gave us confidence on our cleaning procedure to remove the support material trapped into the voids for this range of porosity values. Also, to verify the cleaning process efficiency, we compared the scans of the 3D printed samples before and after the cleaning. Figure 6 illustrates two examples of cleaned pores.

**Figure 7:**
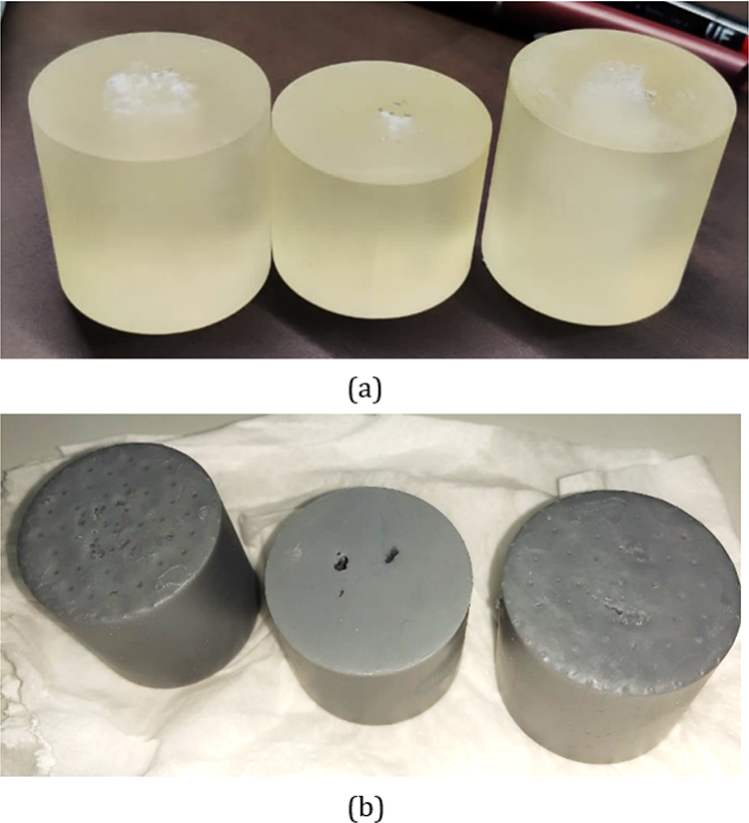
3D printed samples using (a) ProJet MJP 3600 and (b) Form 2.

Based on the results obtained with samples S_5_ and S_6_, we generated new samples with lower porosity values to ensure a range of permeabilities lower than the experimental laboratory setup maximum sensitivity. We selected several X-ray microtomography subset images of Fontainebleau and Berea sandstones in addition to Grossmont carbonate and other carbonate rocks from Abu Dhabi oilfield reservoir rocks (S_5_ to S_10_). Figure 7 shows 3D printed samples generated by using the ProJet MJP 3600 and the Form 2.

We also conducted experimental and numerical tests to estimate the porosity and permeability of cylindrical samples S_7_ to S_10_ (Table 3). We implemented all LBM simulations using the Palabos library. We used 12 nodes with 12 cores each to simulate permeability values from each image of 1000^3^ voxel size. Convergence was obtained in a range of 6 to 10 h depending on the sample. Experimental permeability and porosity values ranged from 1276 to 2780 mD and from 2 to 6%, respectively.

**Table 3:** 3D Printed Real Rock Models Using the ProJet 3D Printer: Experimental and Simulated Rock Properties^a^

			porosity (%)		permeability (mD)	
type		size (mm)	digital	experimental	digital	experimental
S_5_	carbonate	38	12.1	12.3	39,590	NA
S_6_	carbonate	38	11.6	11.5	49,841	NA
S_7_	carbonate	38	3.3	2.8	4168	3780
S_8_	carbonate	12.7	5.2	4.7	2508	1864
S_9_	sandstone	12.7	6.7	6.1	1590	1276
S_10_	sandstone	12.7	4.1	3.5	2304	1943

a Each sample has the same height and diameter sizes.

The comparison between experimental and simulated porosity values shows higher errors than for S_1_ and S_2_ samples. This result was expected as for low porosity samples, pore structures are less connected making the cleaning process more challenging when removing the trapped support material. Indeed, the cleaning process for samples S_7_ to S_10_ reached in some cases two weeks, whereas we could clean the first samples S_1_ to S_6_ in less than a week in general.

**Figure 8:**
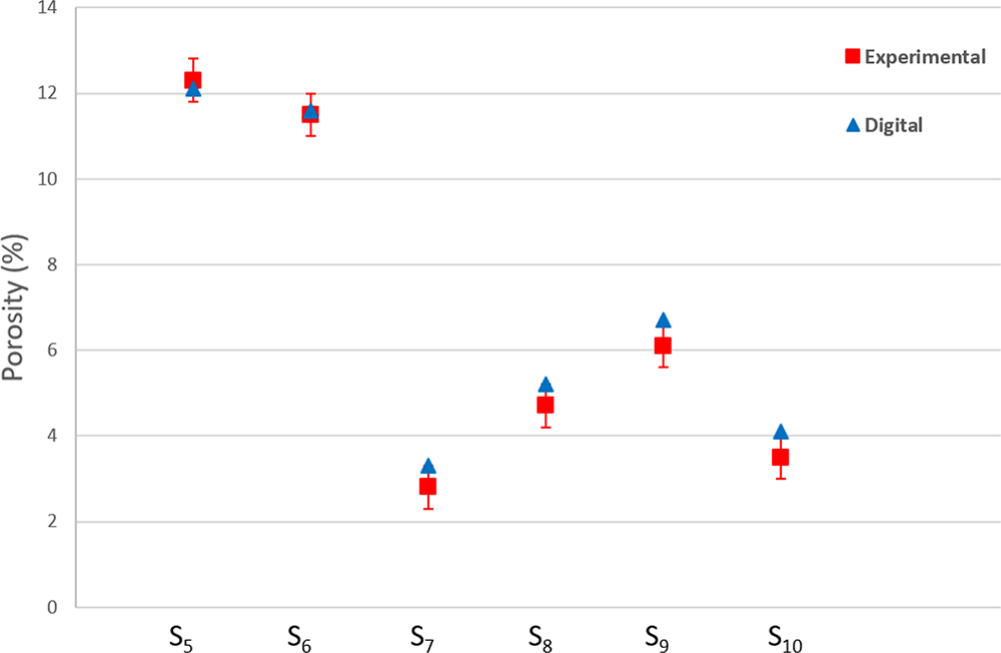
Comparison between digital and experimental porosity values for samples S_5_ to S_10_.

**Figure 9:**
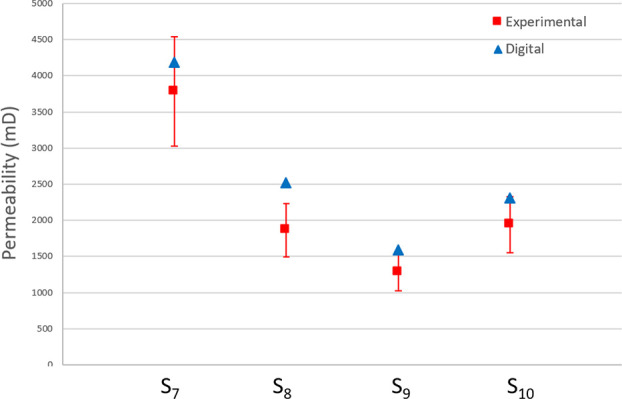
Comparison between digital and experimental permeability values for samples S_7_ to S_10_.

Overall, the experimental porosities are lower than the digital ones, meaning that some of the pores are not efficiently cleaned. Nevertheless, differences between experimental and digital values range from 0.1 to 0.6% (Figure 8), which are relatively low. The nonefficiency of cleaning caused differences between the experimental and digital values. However, these differences are acceptable as they are within the experimental range of errors. Moreover, the experimental and digital permeability values are in the same ranges (Figure 9). The triangulation and smoothing of the meshing, which was applied to create the solid-phase surface, might be another source of error due to some limitations in representing pore connections. For example, we simulated permeability in samples S_7_, S_8_, S_9_, and S_10_ using the original segmented image and found it to be equivalent to the permeability simulation using the surface (stl) file. Nevertheless, some of the pore connections with sizes at the limit of the 3D printer machine resolution were closed during the printing process preventing fluid flow into the entire pore network.

## 4 Conclusions

We studied the feasibility of using 3D printing to magnify subset samples representing porous media scanned with a 3D X-ray microtomography scanner at a high resolution. We used the ProJet MJP 3600 and Form 2 3D printers to replicate porous media. The flush-cleaning method proposed in this study, though time-consuming, is effective. We printed several synthetic and real rock models, and then, we digitally and experimentally estimated their porosity and permeability values. The printing parameters and the type of technology implemented using ProJet MJP 3600 and Form 2 3D printers did not affect significantly the final experimental measurements. Overall, the porosity and absolute permeability estimation methods proposed in this study showed relatively good agreement with analytical and experimental values for the 10 studied samples. Discrepancies are mainly due to difficulties encountered during the cleaning process originating from limitations of the 3D printing resolution and surface meshing representation at the pore connection level. An important outcome of this work is to prove that we can do reliable a one-to-one comparison between experimental and numerical simulations based on the LBM. The results obtained confirm the high reliability of this numerical method even in complex geometry carbonate samples at the pore scale.

## Abbreviations

ADNOC: Abu Dhabi National Oil Company
ADRIC: ADNOC Research and Innovation Center
CFD: computational fluid dynamics
CNN: convolutional neural network
CT: computed tomography
D (or mD): darcy (or millidarcy) (permeability measuring unit)
FDM: finite difference method
FIB-SEM: focused ion beam scanning electron microscopy
IPA: isopropyl alcohol
LBM: lattice Boltzmann method
Palabos: parallel lattice Boltzmann solver
poroperm: porosity and permeability
RAW: file format of unprocessed data
SLA: stereolithography (technology)
STL: stereolithography (file format)
TIF: tagged image file format
U-Net: architecture for semantic segmentation
